# Inhibition of histamine accumulation by novel histamine‐degrading species of *Staphylococcus* sp. isolated from goats and sheep milk

**DOI:** 10.1002/fsn3.2723

**Published:** 2022-01-09

**Authors:** Safoora Pashangeh, Seyed Shahram Shekarforoush, Mahmoud Aminlari, Saeid Hosseinzadeh, Victor Nizet, Samira Dahesh, Samane Rahmdel

**Affiliations:** ^1^ Department of Food Hygiene and Public Health School of Veterinary Medicine Shiraz University Shiraz Iran; ^2^ Department of Biochemistry School of Veterinary Medicine Shiraz University Shiraz Iran; ^3^ Department of Pediatrics Skaggs School of Pharmacy and Pharmaceutical Sciences University of California, San Diego La Jolla California USA

**Keywords:** capillary zone electrophoresis, enterotoxin, histamine degradation, milk, *Staphylococcus*

## Abstract

Histamine is an active amine compound that occurs in various fermented foods that may cause adverse effects on the human health. Certain microorganisms are able to degrade histamine by an oxidative deamination reaction. Therefore, the present study aimed to quantify histamine‐forming and/or ‐degrading activity of the isolates derived from milk of goat and sheep herds, in Iran, by the capillary zone electrophoresis (CZE) method; and we evaluated the molecular characteristics of staphylococcal isolates. Among 243 staphylococcal isolates, 29 histamine‐degrading bacteria were identified. One of these isolates, identified as *Staph. epidermidis*, No. 605, exhibited the highest activity compared to others, degrading available histamine to 58.33% within 24 h. By polymerase chain reaction (PCR) analysis, the isolate, No. 605 that exhibited remarkable histamine‐degrading activity lacked the genes encoding coagulase and DNase, nor did it harbor any of the five classical enterotoxin genes. This is the first report to show that seven *Staphylococcus* species, including *Staph. chromogenes*, *Staph. aureus*, *Staph. haemolyticus*, *Staph. epidermidis*, *Staph*. *pseudintermedius*, *Staph*. *agnetis*, and *Staph. hyicus*, were able to degrade histamine, which were hitherto not known to have this capacity. Therefore, histamine‐degrading activity is a definite criterion to introduce fermenting organisms able to decrease histamine content in different food products.

## Graphical abstract

Among 243 staphylococcal isolates, 29 histamine‐degrading bacteria were identified. One of these isolates, identified as *Staph. epidermidis*, No. 605, exhibited the highest activity compared to others, degrading available histamine to 58.33% within 24 h. By PCR analysis, the isolate, No. 605 that exhibited remarkable histamine‐degrading activity lacked the genes encoding coagulase and DNase, nor did it harbor any of the five classical enterotoxin genes.

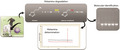

## 1 INTRODUCTION

Histamine is well known as the scombroid poisoning agent and it is considered as a natural antinutrition factor which is very important in food hygiene (Gonzaga et al., 2009). It is present in various levels in red wine and beer, fish, milk and cheese, sausage, salami, vegetables, and many other popular foods and beverages (Lorenzo et al., 2007). Histamine in food is mainly formed by microorganisms which are able to decarboxylate histidine. Histidine decarboxylation is catalyzed by l‐histidine decarboxylase in the conditions proper for bacterial growth and subsequent decarboxylase activity (Rivas et al., 2008). Food proteolysis during processing or storage can produce free histidine or it can be found naturally in foods. Therefore, high levels of histamine in food products are usually associated with microbial fermentation. In this sense, we can consider histamine as an index for food hygiene and quality (Maintz & Novak, 2007). In addition, the presence of high amounts of histamine in food is associated with foodborne disease and can be of health concern. Regarding fish consumption, the U.S. Food and Drug Administration (FDA) suggested that histamine content higher than 200 mg/kg can cause histamine (scombroid) fish poisoning (Lehane & Olley, 2000).

Histamine is physiologically inactivated by histamine oxidase. The oxidative deamination process of histamine is related to the production of hydrogen peroxide, ammonia, and imidazole acetaldehyde (Sekiguchi et al., 2004). This oxidation activity has been characterized in *Staph*. *xylosus*, *Staph*. *carnosus*, *Bacillus amyloliquefaciens*, *Arthrobacter crystallopoietes*, and *Brevibacterium linens* (Martuscelli et al., 2000; Sekiguchi et al., 2004; Zaman et al., 2010). Recently, the main strategy of using bacteria with histamine degradation activity has appeared as an approach for reduction of food histamine content (Mah & Hwang, 2009; Naila et al., 2010). Nevertheless, few reports of staphylococcal strains possessing biogenic amine‐degrading activity have been described. For instance, *Staph. carnosus* FS19 was found to possess histamine oxidase capable of degrading histamine up to 29.1% from its initial concentration within 24 h in laboratory experiments (Zaman et al., 2010, 2014). *Staph*. *xylosus* S81, isolated from artisanal fermented sausages, and *Staph*. *xylosus* isolated from the anchovy, also exhibited histamine degradation activity, degraded the histamine content by about 100% and 38% within 48 h, respectively (Lee et al., 2013; Martuscelli et al., 2000).

This research was conducted to isolate and characterize staphylococcal strains possessing histamine‐degrading activity from milk of goats and sheep. Histamine‐forming and/or ‐degrading activity of the isolates was quantified and molecular characteristics of the isolates described to classify them regarding food safety.

## 2 MATERIALS AND METHODS

### 2.1 Isolation of staphylococcal strains

Milk samples (10 animals/farm) were collected from 50 distinct farms settled in four areas of Fars, Iran. All the farms practiced manual milking. Discarding first streams of milk, 25 ml from both cleaned and disinfected teats was collected in a sterile tube and samples were immediately transferred on ice. Following, samples (10 μl of each) were subcultured on Baird–Parker agar and passed 48 h of incubating at 37°C. Morphological characteristics, gram staining, and catalase and oxidase tests were applied for colony screening. Regarding mentioned properties, colonies representing genus *Staphylococcus* were picked and inoculated in trypticase soy broth (TSB) containing 40% sterile glycerol and stored at −20°C for further tests (Pilipčincová et al., 2010).

### 2.2 Identification of staphylococcal isolates by PCR

The primers used for the identification of staphylococcal strains, Tstag765 and TstaG422, were designed as previously reported (Morot‐Bizot et al., 2004) employing a uniplex polymerase chain reaction (PCR) assay. PCR was performed under the following conditions: 3 min at 94°C, then 40 cycles of 1 s at 95°C, 30 s at 55°C, 30 s at 72°C, and a final hold of 3 min at 72°C with a gradient automated thermocycler (Bioer XP Cycler). The PCR mixture was analyzed by 1.4% agarose gel electrophoresis in Tris‐acetate‐EDTA (TAE) (1×).

### 2.3 Histamine‐degrading activity of isolates

Cells of overnight culture of each isolate were harvested by centrifugation (14,000*g* for 5 min), washed once with phosphate buffer (0.1 M; pH 7.0), and inoculated in 5 ml of sodium phosphate buffer supplemented with histamine (1 mM). The suspension was then incubated at 37°C for 24 h. Histamine‐supplemented medium without bacterial culture was used as negative control, while *Staph*. *xylosus* PTCC 1444 (Iranian Research Organization for Research and Technology [IROST], Iran) functioned as positive control (Mah & Hwang, 2009; Martuscelli et al., 2000). After incubation, the culture was centrifuged at 14,000*g* for 5 min (Eppendorf) at 4°C and the supernatant was filtered with 0.45‐μm filter paper (Lee et al., 2013). One milliliter of the culture broth was taken and frozen at −80°C for quantitation of histamine using capillary zone electrophoresis (CZE) method, as described by Numanoğlu et al. (2008).

### 2.4 Histamine formation activity of isolates

A similar process was performed for histamine formation activity of isolates. Cells of overnight culture of each isolate (adjusted to 2 × 10^8^ CFU/ml) were washed once with phosphate buffer (0.1 M; pH 7.0), pelleted by centrifugation (14,000*g* for 5 min), and inoculated in 5 ml of sodium phosphate buffer supplemented with histidine (0.5 mM) for 24 h incubation at 37°C. Five ml of histidine‐supplemented phosphate buffer (0.5 mM) with no bacterial culture and one incubated with *Staph. epidermidis* TYH1, histamine‐forming strain isolated from fish‐miso in Japan (Yokoi et al., 2011), were used as negative and positive controls, respectively. After incubation, the supernatant was removed by centrifugation (at 14,000*g* for 5 min) at 4°C and filtered through a 0.45‐μm filter (Lee et al., 2013). The supernatants were preserved at −80°C for CZE analysis.

### 2.5 Assay for histamine content (CZE analysis of histamine)

The histamine content was determined by the CZE analysis described by Numanoğlu et al. (2008) with some modification. A CZE apparatus (Prince Autosampler, Model 1‐Lift, 450 Series) was applied for histamine analysis. This system was supplied with a thermometer and a UV‐visible (UV‐vis) detector (set at 25°C and 210 nm, respectively). CZE data were then analyzed by Data Acquisition and Analysis Software, DAx. The separation was accomplished with phosphate buffer (50 mM, pH 2.5) and the injection of samples (hydrodynamically at 50 mbar for 3 s) was performed under constant voltage conditions of 20 KV and normal polarity. The capillary utilized had a proper length of 52 cm and 75 μm of internal diameter (Prince Autosampler). Peak area was used for the determination of histamine in samples.

### 2.6 Molecular characterization of histamine‐degrading staphylococcal isolates

Molecular characteristics of the isolates obtained in the present study were performed by identification of genus and detecting coagulase (*coa*), thermostable nuclease (*nuc*), and staphylococcal enterotoxin (SE) (*sea*, *seb*, *sec*, *sed*, and *see*) genes.

#### 2.6.1 DNA extraction

Genomic DNA was isolated from staphylococcal strains with histamine‐degrading activity, using a DNeasy Blood and Tissue kit (Qiagen GmbH) with modification, based on manufacturer's instructions.

#### 2.6.2 Primers

Table 1 exhibits the nucleotide sequences of all PCR primers applied in this study and their respective amplified products. Primers were synthesized by CinnaGen Co.

**TABLE 1:** Nucleotide sequences and predicted size of polymerase chain reaction (PCR) products for the staphylococcal‐specific oligonucleotide primers

Gene	Primer	Oligonucleotide sequences (5′–3′)	PCR product (bp)	PCR	References
*Tstag*	Tstag422	GGCCGTGTTGAACGTGGTCAAATCA	370	Uniplex	Morot‐Bizot et al. (2004)
	Tstag765	TIACCATTTCAGTACCTTCTGGTAA			
*sea*	SEA‐f	CCTTTGGAAACGGTTAAAACG	127	Multiplex	Barati et al. (2006)
	SEA‐r	TCTGAACCTTCCCATCAAAAAC			
*seb*	SEB‐f	TCGCATCAAACTGACAAACG	477	Multiplex	Barati et al. (2006)
	SEB‐r	GCAGGTACTCTATAAGTGCCTGC			
*sec*	SEC‐f	CTCAAGAACTAGACATAAAAGCTAGG	271	Multiplex	Barati et al. (2006)
	SEC‐r	TCAAAATCGGATTAACATTATCC			
*sed*	SED‐f	CTAGTTTGGTAATATCTCCTTTAAACG	319	Multiplex	Barati et al. (2006)
	SED‐r	TTAATGCTATATCTTATAGGGTAAACATC			
*see*	SEE‐f	CAGTACCTATAGATAAAGTTAAAACAAGC	178	Multiplex	Barati et al. (2006)
	SEE‐r	TAACTTACCGTGGACCCTTC			
*rpo*	RpoB‐1418f	CAATTCATGGACCAAGC	899	Uniplex	Mellmann et al. (2006)
	RpoB‐3554r	CCGTCCCAAGTCATGAAAC			
*nuc*	Nuc‐f	GCGATTGATGGTGATACGGTT	270–300	Uniplex	Brakstad et al. (1992)
	Nuc‐r	AGCCAAGCCTTGACGAACTAAAGC			
*coa*	coa‐f	CGAGACCAAGATTCAACAAG	Variable	Uniplex	Raimundo et al. (1999)
	coa‐r	AAAGAAAACCACTCACATCA			

#### 2.6.3 Uniplex PCR

All the uniplex PCRs employed in the present study were set up in a final volume of 25 μl, containing 50–100 ng genomic DNA, 0.1 µM of the respective primer and 12.5 μl of Taq DNA polymerase 2.0× Master Mix RED (1.5 mM MgCl_2_; Ampliqon). The amplification was carried out in a gradient automated thermalcycler with a hot bonnet (Bioer XP Cycler) and analyzed by 1.4% agarose gel electrophoresis in TAE (1×).

#### 2.6.4 Identification of *Staphylococcus* genus

The identities of histamine‐degrading isolates were further confirmed by amplifying and sequencing a single 899 bp band of *rpoB* gene, encoding the beta subunit of RNA polymerase, using primers described by Mellmann et al. (2006). Amplifications were carried out as follows: initial denaturation 94°C for 5 min followed by 35 cycles of denaturation (94°C for 45 s), annealing (52°C for 1 min), elongation (72°C for 90 s), and then a final elongation at 72°C for 10 min (Mellmann et al., 2006). The amplified genes were finally extracted from gels using QIAquick PCR Purification Kit (Qiagen), as described by the manufacturer. The pure products were subjected to sequencing (Macrogen). Identification of histamine‐degrading bacteria was approved by sequence analyzing, using the Basic Local Alignment Search Tool (BLAST) (National Center for Biotechnology Information [NCBI]).

#### 2.6.5 Detection of the coagulase gene

All *Staphylococcus* isolates were subjected to PCR cycles for coa gene (Table 1) (Raimundo et al., 1999), consisting of preheating at 94°C for 45 s, denaturation at 94°C for 20 s, annealing at 57°C for 15 s, and extension at 70°C for 15 s for 30 times. The amplification was carried out with a final extension step at 72°C for 2 min, and the isolates were stored at 4°C until the products were collected. The PCR products were detected by electrophoresis in a 1.4% agarose gel in TAE (1×), as previously described (Ahmadi et al., 2010).

#### 2.6.6 Detection of deoxyribonuclease gene

The PCR cycles for the *nuc* gene consisted of thermal cycles of 94°C for 1 min, 55°C for 30 s, and 72°C for 90 s and were repeated 37 times. The amplifications were performed with a final extension step at 72°C for 2 min, and the isolates were stored at 4°C, until the products were collected. The PCR products were detected by electrophoresis in a 1.4% agarose gel in TAE (1×) (Brakstad et al., 1992).

#### 2.6.7 Detection of classical enterotoxin genes by multiplex PCR

The presence of classical enterotoxin genes, sea, seb, sec, sed, and see (Table 1; Barati et al., 2006) among the *Staphylococcus* isolates obtained, was investigated employing a multiplex PCR assay as previously described by Omoe et al. (2005). The reaction mixture (50 μl) containing 0.1 µM of each primer, 50–100 ng genomic DNA, and 25 μl of Taq DNA polymerase 2.0× Master Mix RED was used (1.5 mM MgCl2; Ampliqon). *Staph*. *aureus* reference strains, *Staph*. *aureus* DSM 19,040 (SEC, SEE) and *Staph*. *aureus* DSM 19,041 (SEA, SEB, SED), were used as enterotoxin producers (Rahmdel et al., 2018). The products of PCR were detected by electrophoresis in a 1.4% agarose gel in TAE (1×).

### 2.7 Statistical analysis

All the experiments were carried out in triplicate and the results were expressed as mean values and standard deviations. Data analyses were performed using SPSS software Version 16.0 for Windows. The mean comparison was performed using the Duncan's Multiple Range Test (DMRT) at *p* < .05 significant difference following analysis of variance (ANOVA).

## 3 RESULTS AND DISCUSSION

Based on biochemical and morphological characteristics, we differentiated colonies that represented staphylococcal isolates. With the help of Baird–Parker selective medium, 500 staphylococcal colonies were differentiated. Typical jet black colonies were observed in all the samples screened on Baird–Parker agar plates, and a subset of them revealed colonies surrounded by a clear opaque zone or halo. All 500 isolates showed typical Gram‐positive staining, morphological characteristics of cocci in clusters, and were also positive for catalase and negative for oxidase activity. The PCR amplification of the *Tstag* gene resulted in a single 370 bp product in 243 of the screened isolates.

For histamine‐degrading ability, we inoculated resting cells of these 243 different confirmed staphylococcal isolates in 5 ml of sodium phosphate buffer containing 1 mM histamine for 24 h, and Table 2 lists the number of 29 histamine‐degrading strains, determined by the CZE method. The histamine levels obtained in the samples were within the standard value (0.05–1.78 mM). The calibration graph was linear in a range of 0.05–1.78 mM with a regression equation, *y* = 0.000004*x* (*r* ^2^ = .999). As shown in Table 2, isolate No. 605 exhibited a significantly greater ability to degrade histamine, to 58.33% within 24 h, and was subsequently identified as *Staph. epidermidis*. The other isolates tested had a range of noticeable effects in degrading histamine. Our positive control, *Staph. xylosus*, degraded 17.70% of initiate histamine content, whereas the negative control exhibited no histamine degradation.

**TABLE 2:** Identification of histamine‐degrading *Staphylococci* isolated from sheep and goats milk by capillary zone electrophoresis (CZE) and histamine degradation by the isolates in sodium phosphate buffer (pH 7.0) supplemented with 1 mM histamine after incubation at 37°C for 24 h

Strain no	Strain species	Histamine residual (mM)^a^	Histamine degradation (%)
–	No bacteria^b^	0.96 ± 0.0002^H^	0
PTCC 1444^c^	*xylosus*	0.79 ± 0.0001^E^	17.70
605	*epidermidis*	0.40 ± 0.0002^A^	58.33
1	*hyicus*	0.52 ± 0.0003^B^	45.83
35	*epidermidis*	0.55 ± 0.0012^B^	42.70
31	*epidermidis*	0.60 ± 0.0008^C^	37.50
55	*haemolyticus*	0.60 ± 0.0012^C^	37.50
17	*chromogenes*	0.61 ± 0.0018^C^	36.45
68	*aureus*	0.62 ± 0.0002^C^	35.41
330	*chromogenes*	0.62 ± 0.0010^C^	35.41
158	*haemolyticus*	0.64 ± 0.0008^C^	33.33
332	*chromogenes*	0.65 ± 0.0001^C^	32.29
95	*aureus*	0.66 ± 0.0006^C^	31.25
53	*haemolyticus*	0.68 ± 0.0001^D^	29.16
13	*rostri*	0.69 ± 0.0002^D^	28.12
211	*chromogenes*	0.69 ± 0.0012^D^	28.12
85	*epidermidis*	0.70 ± 0.0003^D^	27.08
156	*chromogenes*	0.72 ± 0.0008^D^	25.00
29	*aureus*	0.73 ± 0.0006^D^	23.95
354	*pseudintermedius*	0.74 ± 0.0012^D^	22.91
91	*chromogenes*	0.74 ± 0.0003^D^	22.91
344	*aureus*	0.75 ± 0.0001^D^	21.87
65	*chromogenes*	0.76 ± 0.0009^D^	20.83
282	*chromogenes*	0.78 ± 0.0015^E^	18.75
92	*aureus*	0.79 ± 0.0009^E^	17.70
102	*aureus*	0.79 ± 0.0003^E^	17.70
355	*agnetis*	0.83 ± 0.0006^F^	13.54
22	*aureus*	0.84 ± 0.0012^F^	12.50
106	*haemolyticus*	0.85 ± 0.0009^F^	11.45
80	*aureus*	0.85 ± 0.0020^F^	11.45
266	*chromogenes*	0.90 ± 0.0012^G^	6.25

^a^ The numbers represent the mean ± standard deviation of three determinations. Values followed by different letters in the same column are significantly different (*p* < .05).

^b^ Negative control.

^c^ Positive control.

In this study, nine of 29 histamine‐degrading isolates (31.0%) from milk of goats and sheep belonged to *Staph. chromogenes*, eight (27.6%) to *Staph. aureus*, four (13.8%) to *Staph. haemolyticus*, four (13.8%) to *Staph. epidermidis*, one (3.4%) to *Staph*. *pseudintermedius*, one (3.4%) to *Staph*. *agnetis*, one (3.4%) to *Staph. hyicus*, and one (3.4%) to *Staph*. *rostri*. The most potent histamine‐degrading isolate detected was *Staph. epidermidis* (isolate No. 605). The observation that histamine‐degrading ability varied considerably across *Staphylococcus* of different species is consistent as per reports from several authors regarding diversity in this key phenotype. Monoamine oxidase and histamine degradation activities were observed by most of the *Staph. xylosus* strains tested in the study conducted by Martuscelli et al. (2000). The most histamine‐degrading activity of *Staph*. *xylosus* strains was observed after incubation of 48 h. Similarly, *Staph. carnosus* FS19 derived from fish sauce reduced histamine content up to 29.1% from its initial concentration within 24 h (Zaman et al., 2010, 2011). In addition, some strains of *Staphylococcus* were reported for their histamine‐degrading enzymes (Mah & Hwang, 2009). *Staph. xylosus* No. 0538 exhibited the maximum ability for histamine degradation, 38.0% of the histamine content (0.5 mM in phosphate buffer) after an incubation time of 24 h. Zaman et al. (2014) demonstrated that *Staph*. *carnosus* FS19 was able to degrade 15.1% and 13.8% of histamine content. *Staph. xylosus* S81 and S142, isolated from artisanal fermented sausages, and *Staph. xylosus*, isolated from the anchovy, showed histamine degradation activity (Lee et al., 2013; Martuscelli et al., 2000). Recently, Sun et al. (2020) investigated the effect of different starter cultures on biogenic amines’ content in a kind of fermented sausage. They observed that *Staph. pentosans* exhibited oxidase activity and was able to reduce histamine content. Although *Staph. chromogenes*, *Staph. aureus*, *Staph. haemolyticus*, *Staph. epidermidis*, *Staph*. *pseudintermedius*, *Staph*. *agnetis*, and *Staph*. *hyicus* were not previously reported as histamine‐degraders, they accounted for the histamine‐degrading isolates in this study. This is the first report to show that other staphylococcal strains other than *Staph. xylosus* and *Staph. carnosus* are able to degrade histamine.

Histamine formation activities of 29 isolates, which exhibited histamine‐degrading ability, were tested by inoculating resting cells in 5 ml of sodium phosphate buffer containing 0.5 mM histidine. Isolate No. 53 was the only one with histamine‐forming activity, forming histamine to about 0.052 ± 0.0002 mM after incubation of 24 h at 37°C, and was identified as *Staph. haemolyticus*, also possessing histidine decarboxylase activity. This isolate also degraded 29.16% of initiate histamine content in our assay. All amine oxidase‐positive strains showed simultaneous amino acid decarboxylase activity (Voigt & Eitenmiller, 1978). Out of other isolates from our collection tested, 29 isolates, which exhibited histamine‐degrading ability, did not show histamine‐forming activity. The positive control, *Staph. epidermidis* TYH1, and negative control exhibited 0.41 ± 0.0003 and 0.0 mM histamine content, respectively. According to the histamine‐forming ability of *Staphylococcus* strains isolated from various foods, Simonova et al. (2006) reported that *Staph. carnosus* SO2/F/2/5, isolated from Slovak traditional meat products, was the only one strain which exhibited production of the biogenic amine. Several authors (Bover‐Cid et al., 2001; Casaburi et al., 2005; Karovicova & Kohajdova, 2005) reported similar results of biogenic amine production by *Staphylococcus* strains.

Different methods have been used to analyze the histamine content of food (Bartkiene et al., 2020; Lange & Wittmann, 2002; Lapa‐Guimaraes & Pickova, 2004; Tang et al., 2020). The application of CZE for the determination of biogenic amines (including histamine) has been described in a variety of food products in many studies (Er et al., 2014; Kvasnicka & Voldrich, 2006; Sun et al., 2003; Vitali et al., 2013).

**FIGURE 1:**
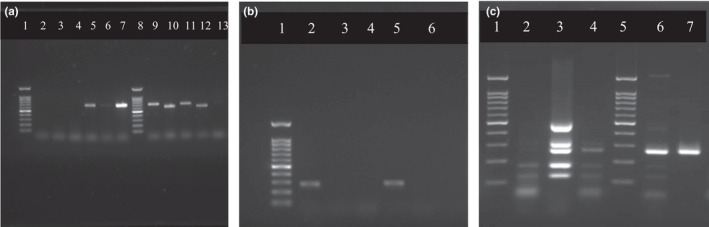
(a) Electrophoretic agarose gel images of *coa* gene polymerase chain reaction (PCR) products of histamine‐degrading staphylococcal strains. Lane 1, 8:100 bp ladder, lane 5, 7, 9, 10, 11, 12: positive strains, lane 13: negative control. (b) Electrophoretic agarose gel images of *nuc* gene PCR products of histamine‐degrading staphylococcal strains. Lane 1:100 bp ladder, lane 2, 5: positive strains, lane 6: negative control. (c) Electrophoretic agarose gel images of multiplex PCR amplification of classical staphylococcal enterotoxin (SE) genes in histamine‐degrading staphylococcal strains. Lane 1, 5:100 bp ladder, lane 3: positive control, lane 6, 7: staphylococcal enterotoxin type C (SEC)‐specific amplicon

The results of molecular identification of histamine‐degrading staphylococcal isolates are shown in Table 3. Out of 29 staphylococcal isolates, the *coa* gene was obtained in 15 strains (51.7%), including 8 *Staph. aureus*, 2 *Staph. chromogenes*, 2 *Staph. epidermidis*, 1 *Staph. pseudintermedius*, 1 *Staph. hyicus*, and 1 *Staph. haemolyticus*. Other isolates were PCR‐negative for the *coa* gene (Figure 1a; Table 3). In our study, the *coa* gene appeared as one of the two different‐sized amplicons, viz., 603 and 720 bp. All isolates were observed to produce only one type of amplicon, either 603 or 720 bp.

**TABLE 3:** Results of testing *Staphylococci* isolates for *coa*, *nuc* and classical enterotoxin genes derived from the agarose gel analysis of uniplex and multiplex polymerase chain reactions (PCRs)

Strain no	Strain species	Molecular characterization		
		*Coa* ^a^	*Nuc* ^b^	SE^c^
605	*epidermidis*	−	−	−
1	*hyicus*	+	+	A,E
35	*epidermidis*	−	+	A
31	*epidermidis*	+	+	A,C
55	*haemolyticus*	−	+	A,C,E
17	*chromogenes*	−	−	−
68	*aureus*	+	+	C
330	*chromogenes*	−	+	C
158	*haemolyticus*	−	+	A,B,C,D,E
332	*chromogenes*	−	+	A,C
95	*aureus*	+	+	C
53	*haemolyticus*	+	+	C
13	*rostri*	−	−	A,E
211	*chromogenes*	+	+	C
85	*epidermidis*	+	−	−
156	*chromogenes*	−	−	C
29	*aureus*	+	+	C,E
354	*pseudintermedius*	+	+	A,B,C,E
91	*chromogenes*	+	+	C
344	*aureus*	+	+	C
65	*chromogenes*	−	+	A,C,E,D
282	*chromogenes*	−	−	C
92	*aureus*	+	+	C,E
102	*aureus*	+	+	C
355	*agnetis*	−	+	B,C
22	*aureus*	+	+	A,B
106	*haemolyticus*	−	−	C
80	*aureus*	+	+	A,B
266	*chromogenes*	−	−	C

Note

+, positive; −, negative.

^a^ Coagulase.

^b^ DNase.

^c^ S*taphylococcus* enterotoxin.

Amplification with *nuc* primers revealed that out of 29 histamine‐degrading isolates, 21 were positive (72.4%) for the amplification of a 270–300 bp specific band (Figure 1b), corresponding to the *nuc* gene, including 8 strains identified as *Staph. aureus*, 5 *Staph. chromogenes*, 3 *Staph. haemolyticus*, 2 *Staph. epidermidis*, 1 *Staph. pseudintermedius*, 1 *Staph. hyicus*, and 1 *Staph. agnetis*. Eight isolates were *nuc* PCR‐negative strains.

One of the important factors determining the pathogenicity of *Staphylococcus* is their ability to produce enterotoxins (SEs). SEs are commonly produced by coagulase‐positive *Staphylococcus* sp. but reports of detection of SE genes among coagulase‐negative *Staphylococcus* are also available. Currently, 23 different SEs are known but the 5 classical SEs especially SE type A (SEA) and SE type D (SED) are responsible for causing more than 95% of staphylococcal food poisoning cases (Podkowik et al., 2013). In the present study, multiplex PCR‐based screening of the 29 *Staphylococcus* strains for classical enterotoxin genes revealed the presence of all SEs among at least a subset of isolates (Figure 1c). The SE type C (SEC) enterotoxin was the most commonly detected enterotoxin gene among the isolates followed by SEA, SE type E (SEE), SE type B (SEB), and SED genes. Twenty‐one isolates of staphylococcal strains revealed the presence of SEC (72.4%), 11 isolates were positive for SEA (37.9%). SEE (27.6%), SEB (17.2%), and SED (6.9%) were positive in 8, 5, and 2 isolates, respectively, while 3 isolates did not reveal the presence of any of these five classical enterotoxin genes (10.3%). No significant relation was observed between staphylococcal isolates containing enterotoxin gene with histamine‐forming and/or ‐degrading activity of the isolates. Isolate No. 605, *Staph. epidermidis*, which exhibited a remarkable histamine‐degrading activity did not harbor any of the five classical enterotoxin genes. The only one histamine‐forming isolate, No. 53, identified as *Staph. haemolyticus*, was positive for SEC. Out of the 26 SE positive isolates, 12 were coagulase‐negative *Staphylococcus* (46.1%) while the remaining 14 were coagulase‐positive (53.8%). Recently, Podkowik et al. (2013) reviewed the enterotoxigenic potential of coagulase‐negative staphylococci and its role in staphylococcal food poisoning. Production of enterotoxin by coagulase‐negative *Staphylococcus* was also reported by other investigators (Rall et al., 2010). Collery et al. (2008) showed that of the classical enterotoxin genes, SEA, SEB, and SEC were detected commonly among *Staph*. *aureus* with SEB the most common, which was likewise observed in our study.

## 4 CONCLUSIONS

Among the 29 histamine‐degrading bacteria isolated from goats and sheep milk, isolate No. 53, identified as *Staph. haemolyticus*, was the only one with histamine‐forming activity. This is the first report to suggest that seven staphylococcal strains, including *Staph. chromogenes*, *Staph. aureus*, *Staph. haemolyticus*, *Staph. epidermidis*, *Staph*. *pseudintermedius*, *Staph*. *agnetis*, and *Staph. hyicus* were able to degrade histamine to a remarkable content. As much as 51.7% of staphylococcal strains with histamine degradation ability were coagulase‐positive, 72.4% *nuc*‐PCR‐positive, and 89.6% of these histamine‐degrading strains were able to produce 1–5 of classical enterotoxins. Concerning the safety aspect of strains by detection of *coa*, *nuc* and enterotoxin genes, we have two strains of *Staph*. *epidermidis* and *Staph*. *chromogenes*, which did not reveal the presence of *coa*, *nuc*, and any of the classical enterotoxin genes, but exhibited a noticeable histamine degradation activity, 58.33% and 36.45% of initiate histamine content within 24 h, respectively. This study suggests that a variety of *Staphylococcus* sp., isolated from milk of goats and sheep, have histamine‐degrading ability, a point of particular interest, both from the point of food safety and quality.

Hence, the finding of this research indicated that, within the staphylococcal strains isolated from milk of goats and sheep, 29 possessed the potential to degrade histamine from 6.25% to 58.33%. However, bacterial histamine oxidase activity in a simple medium of phosphate buffer will be different from their behavior in complex substances, as well as in different incubation times. We observed the histamine degradation amount, two times more than that reported by Zaman et al. (2010), after an incubation time of 24 h. Therefore, the effects of the complexity of culture media and different incubation times on the bacterial histamine degradation should be explored in further researches.

## CONFLICT OF INTEREST

None.

## AUTHOR CONTRIBUTIONS

**Safoora Pashangeh:** Conceptualization (equal); Data curation (equal); Formal analysis (lead); Investigation (lead); Methodology (equal); Project administration (lead); Resources (equal); Software (equal); Writing – original draft (lead); Writing – review & editing (lead). **Seyed Shahram Shekarforoush:** Conceptualization (equal); Data curation (equal); Formal analysis (equal); Funding acquisition (lead); Investigation (equal); Methodology (equal); Project administration (lead); Software (equal); Supervision (lead); Validation (lead); Visualization (lead); Writing – original draft (equal); Writing – review & editing (equal). **Mahmoud Aminlari:** Conceptualization (equal); Data curation (equal); Investigation (supporting); Methodology (equal); Project administration (supporting); Supervision (equal); Validation (equal); Visualization (equal). **Saeid Hosseinzadeh:** Conceptualization (supporting); Data curation (supporting); Funding acquisition (supporting); Investigation (supporting); Methodology (supporting); Project administration (supporting); Supervision (equal); Validation (equal); Visualization (supporting). **Victor Nizet:** Conceptualization (equal); Data curation (supporting); Methodology (supporting); Project administration (supporting); Validation (equal); Writing – original draft (supporting). **Samira Dahesh:** Data curation (supporting); Software (supporting); Writing – original draft (supporting); Writing – review & editing (supporting). **Samane Rahmdel:** Conceptualization (supporting); Investigation (supporting); Methodology (supporting); Validation (supporting).

## Data Availability

The additional data will be available upon requesting the corresponding author.

## References

[fsn32723-bib-0001] Ahmadi, M. , Razavi Rohani, S. M. , & Ayremlou, N. (2010). Detection of *Staphylococcus aureus* in milk by PCR. Comparative Clinical Pathology, 19, 91–94. 10.1007/s00580-009-0901-0

[fsn32723-bib-0002] Barati, B. , Saadati, M. , & Bahmani, M. K. (2006). Isolation and detection of enterotoxigenic *Staphylococcus aureus* type A by multiplex PCR. Military Medicine, 8, 119–128.

[fsn32723-bib-0003] Bartkiene, E. , Mozuriene, E. , Lele, V. , Zokaityte, E. , Gruzauskas, R. , Jakobsone, I. , Juodeikiene, G. , Ruibys, R. , & Bartkevics, V. (2020). Changes of bioactive compounds in barley industry by‐products during submerged and solid state fermentation with antimicrobial *Pediococcus acidilactici* strain LUHS29. Food Sciences and Nutrition, 8(1), 340–350. 10.1002/fsn3.1311 PMC6977520 31993160

[fsn32723-bib-0004] Bover‐Cid, S. , Hugas, M. , Izquierdo‐Polido, M. , & Vidal‐Carou, M. (2001). Amino acid decarboxylase activity of bacteria isolated from fermented pork sausages. International Journal of Food Microbiology, 66, 185–189. 10.1016/S0168-1605(00)00526-2 11428577

[fsn32723-bib-0005] Brakstad, O. G. , Aasbakk, K. , & Maeland, J. A. (1992). Detection of *Staphylococcus aureus* by polymerase chain reaction amplification of the *nuc* gene. Journal of Clinical Microbiology, 30, 1654–1660. 10.1128/jcm.30.7.1654-1660.1992 1629319 PMC265359

[fsn32723-bib-0006] Casaburi, A. , Blaiotta, G. , Mauriello, G. , Pepe, O. , & Villani, F. (2005). Technological activities of *Staphylococcus carnosus* and *Staphylococcus simulans* strains isolated from fermented sausages. Meat Science, 71, 643–650. 10.1016/j.meatsci.2005.05.008 22061209

[fsn32723-bib-0007] Collery, M. M. , Smyth, D. S. , Twohig, J. M. , Shore, A. C. , Coleman, D. C. , & Smyth, C. L. (2008). Molecular typing of nasal carriage isolates of *Staphylococcus aureus* from an Irish university student population based on toxin gene PCR, *agr* locus types and multiple locus, variable number tandem repeat analysis. Journal of Medical Microbiology, 57, 348–358. 10.1099/jmm.0.47734-0 18287299

[fsn32723-bib-0008] De Las Rivas, B. , González, R. , Landete, J. M. , & Muñoz, R. (2008). Characterization of a second ornithine decarboxylase isolated from *Morganella morganii* . Journal of Food Protection, 71(3), 657–661. 10.4315/0362-028X-71.3.657 18389719

[fsn32723-bib-0009] Er, B. , Demirhan, B. , Bas, S. Y. , Yentur, G. , & Oktem, A. B. (2014). Determination of histamine level in canned tuna fish. Bulgarian Journal of Agricultural Science, 20, 834–838.

[fsn32723-bib-0011] Gonzaga, V. E. , Lescano, A. G. , Huamán, A. A. , Salmón‐Mulanovich, G. , & Blazes, D. L. (2009). Histamine levels in fish from markets in Lima, Peru. Journal of Food Protection, 72, 1112–1115. 19517744 10.4315/0362-028x-72.5.1112 PMC4066727

[fsn32723-bib-0012] Karovicova, J. , & Kohajdova, Z. (2005). Biogenic amines in food. ChemInform, 59, 70–79. 10.1002/chin.200534338

[fsn32723-bib-0013] Kvasnicka, F. , & Voldrich, M. (2006). Determination of biogenic amines by capillary zone electrophoresis with conductometric detection. Journal of Chromatography A, 1103, 145–149. 10.1016/j.chroma.2005.11.005 16310200

[fsn32723-bib-0015] Lange, J. , & Wittmann, C. H. (2002). Enzyme sensor array for the determination of biogenic amines in food samples. Analytical and Bioanalytical Chemistry, 372, 276–283. 10.1007/s00216-001-1130-9 11936099

[fsn32723-bib-0016] Lapa‐Guimaraes, J. , & Pickova, J. (2004). New solvent systems for thin‐layer chromatographic determination of nine biogenic amines in fish and squid. Journal of Chromatography A, 1045, 223–232. 10.1016/j.chroma.2004.06.014 15378899

[fsn32723-bib-0017] Lee, J. M. , Lee, D. C. , & Kim, S. M. (2013). The effect of Koji and histidine on the formation of histamine in Anchovy sauce and the growth inhibition of histamine degrading bacteria with preservatives. American Journal of Advanced Food Science and Technology, 1, 25–36.

[fsn32723-bib-0018] Lehane, L. , & Olley, J. (2000). Histamine fish poisoning revisited. International Journal of Food Microbiology, 58, 1–37. 10.1016/S0168-1605(00)00296-8 10898459

[fsn32723-bib-0019] Lorenzo, J. M. , Martinez, S. , Franco, I. , & Carballo, J. (2007). Biogenic amine content during the manufacture of dry‐cured lacón, a Spanish traditional meat product: Effect of some additives. Meat Science, 77(2), 287–293. 22061602 10.1016/j.meatsci.2007.03.020

[fsn32723-bib-0020] Mah, J. H. , & Hwang, H. J. (2009). Inhibition of biogenic amine formation in a salted and fermented anchovy by *Staphylococcus xylosus* as a protective culture. Food Control, 20, 796–801. 10.1016/j.foodcont.2008.10.005

[fsn32723-bib-0021] Maintz, L. , & Novak, N. (2007). Histamine and histamine intolerance. American Journal of Clinical Nutrition, 85, 1185–1196. 10.1093/ajcn/85.5.1185 17490952

[fsn32723-bib-0022] Martuscelli, M. , Crudele, M. A. , Gardini, F. , & Suzzi, G. (2000). Biogenic amine formation and oxidation by *Staphylococcus xylosus* strains from artisanal fermented sausages. Letters in Applied Microbiology, 31, 228–232. 10.1046/j.1365-2672.2000.00796.x 10972734

[fsn32723-bib-0023] Mellmann, A. , Becker, K. , Von Eiff, C. , Keckevoet, U. , Schumann, P. , & Harmsen, D. (2006). Sequencing and staphylococci identification. Emerging Infectious Diseases, 12, 333–336. 10.3201/eid1202.050962 16494767 PMC3373113

[fsn32723-bib-0024] Morot‐Bizot, S. C. , Talon, R. , & Leroy, S. (2004). Development of a multiplex PCR for the identification of *Staphylococcus* genus and four staphylococcal species isolated from food. Journal of Applied Microbiology, 97, 1087–1094. 10.1111/j.1365-2672.2004.02399.x 15479426

[fsn32723-bib-0025] Naila, A. , Flint, S. , Fletcher, G. , Bremer, P. , & Meerdink, G. (2010). Control of biogenic amines in food‐existing and emerging approaches. Journal of Food Science, 75, 139–150. 10.1111/j.1750-3841.2010.01774.x PMC2995314 21535566

[fsn32723-bib-0026] Numanoğlu, E. , Boyaci, I. H. , & Topcu, A. (2008). Simple determination of histamine in cheese by capillary electrophoresis with diode array detection. Journal of Food and Drug Analysis, 16, 74–80. 10.38212/2224-6614.2315

[fsn32723-bib-0027] Omoe, K. , Hu, D. L. , Takahashi‐Omoe, H. , Nakane, A. , & Shinagawa, K. (2005). Comprehensive analysis of classical and newly described staphylococcal superantigenic toxin genes in *Staphylococcus aureus* isolates. FEMS Microbiology Letters, 246, 191–198. 15899405 10.1016/j.femsle.2005.04.007

[fsn32723-bib-0028] Pilipčincová, I. , Bhide, M. , Dudriková, E. , & Trávniček, M. (2010). Genotypic characterization of coagulase‐negative staphylococci isolated from sheep milk in Slovakia. Acta Veterinaria Brno, 79, 269–275. 10.2754/avb201079020269

[fsn32723-bib-0029] Podkowik, M. , Park, J. , Seo, K. S. , Bystroń, J. , & Bania, J. (2013). Enterotoxigenic potential of coagulase‐negative staphylococci. International Journal of Food Microbiology, 163, 34–40. 10.1016/j.ijfoodmicro.2013.02.005 23500613 PMC6671284

[fsn32723-bib-0030] Rahmdel, S. , Hosseinzadeh, S. , Shekarforoush, S. S. , Torriani, S. , Gatto, V. , & Pashangeh, S. (2018). Safety hazards in bacteriocinogenic *Staphylococcus* strains isolated from goat and sheep milk. Microbial Pathogenesis, 116, 100–108. 10.1016/j.micpath.2018.01.016 29355699

[fsn32723-bib-0031] Raimundo, O. , Deighton, M. , Capstick, J. , & Gerraty, N. (1999). Molecular typing of *Staphylococcus aureus* of bovine origin by polymorphism of the coagulase gene. Veterinary Microbiology, 66, 275–284. 10384888 10.1016/s0378-1135(99)00020-6

[fsn32723-bib-0032] Rall, V. L. M. , Sforcin, J. M. , Augustini, V. C. M. , Watanabe, M. T. , Fernandes, J. A. , Rall, R. , Silva, M. G. , & Araújo, J. J. P. (2010). Detection of enterotoxin genes of *Staphylococcus* SP isolated from nasal cavities and hands of food handlers. Brazilian Journal of Microbiology, 41, 59–65. 10.1590/S1517-83822010000100011 24031464 PMC3768627

[fsn32723-bib-0033] Sekiguchi, Y. , Makita, H. , Yamamura, A. , & Matsumoto, K. (2004). A thermostable histamine oxidase from *Arthrobacter crystallopoietes* KAIT‐B‐007. Journal of Bioscience and Bioengineering, 97, 104–110. 10.1016/S1389-1723(04)70176-0 16233600

[fsn32723-bib-0034] Simonova, M. , Strompfova, V. , Marcinakova, M. , Laukova, A. , Vesterlund, S. , Moratalla, M. L. , Bover‐Cid, S. , & Vidal‐Carou, C. (2006). Characterization of *Staphylococcus carnosus* isolated from Slovak meat products. Meat Science, 73, 559–564. 22062552 10.1016/j.meatsci.2006.02.004

[fsn32723-bib-0035] Sun, X. , Du, B. , Zhao, L. , Jin, Y. , Su, L. , Tian, J. , & Wu, J. (2020). The effect of different starter cultures on biogenic amines and quality of fermented mutton sausages stored at 4 and 20°C temperatures. Food Sciences and Nutrition, 8(8), 4472–4483. 10.1002/fsn3.1748 PMC7455928 32884727

[fsn32723-bib-0036] Sun, X. , Yang, X. , & Wang, E. (2003). Determination of biogenic amines by capillary electrophoresis with pulsed amperometric detection. Journal of Chromatography A, 1005, 189–195. 10.1016/S0021-9673(03)00927-0 12924793

[fsn32723-bib-0037] Tang, H. , Darwish, W. S. , El‐Ghareeb, W. R. , Al‐Humam, N. A. , Chen, L. , Zhong, R. M. , Xiao, Z. J. , & Ma, J. K. (2020). Microbial quality and formation of biogenic amines in the meat and edible offal of *Camelus dromedaries* with a protection trial using gingerol and nisin. Food Sciences and Nutrition, 8(4), 2094–2101. 10.1002/fsn3.1503 PMC7174210 32328276

[fsn32723-bib-0038] Vitali, L. , Valese, A. C. , Azevedo, M. S. , Gonzaga, L. V. , Costa, A. C. O. , Piovezan, M. , Vistuba, J. P. , & Micke, G. A. (2013). Development of a fast and selective separation method to determine histamine in tuna fish samples using capillary zone electrophoresis. Talanta, 106, 181–185. 10.1016/j.talanta.2012.12.020 23598114

[fsn32723-bib-0039] Voigt, M. N. , & Eitenmiller, R. R. (1978). Role of histidine and tyrosine decarboxylases and mono‐diamine oxidases in amine build‐up in cheese. Journal of Food Protection, 41, 182–186. 30795038 10.4315/0362-028X-41.3.182

[fsn32723-bib-0040] Yokoi, K. , Harada, Y. , Shozen, K. I. , Satomi, M. , Taketo, A. , & Kodaira, K. I. (2011). Characterization of the histidine decarboxylase gene of *Staphylococcus epidermidis* TYH1 coded on the staphylococcal cassette chromosome. Gene, 477, 32–41. 10.1016/j.gene.2011.01.003 21236322

[fsn32723-bib-0041] Zaman, M. Z. , Abu Bakar, F. , Jinap, S. , & Bakar, J. (2011). Novel starter cultures to inhibit biogenic amines accumulation during fish sauce fermentation. International Journal of Food Microbiology, 145, 84–91. 10.1016/j.ijfoodmicro.2010.11.031 21183239

[fsn32723-bib-0042] Zaman, M. Z. , Abu Bakar, F. , Selamat, J. , Bakar, J. , See Ang, S. , & Chong, C. Y. (2014). Degradation of histamine by the halotolerant *Staphylococcus carnosus* FS19 isolate obtained from fish sauce. Food Control, 40, 58–63. 10.1016/j.foodcont.2013.11.031

[fsn32723-bib-0043] Zaman, M. Z. , Bakar, F. A. , Selamat, J. , & Bakar, J. (2010). Occurrence of biogenic amines and amines degrading bacteria in fish sauce. Czech Journal of Food Sciences, 28, 440–449. 10.17221/312/2009-CJFS

